# PTools: an opensource molecular docking library

**DOI:** 10.1186/1472-6807-9-27

**Published:** 2009-05-01

**Authors:** Adrien Saladin, Sébastien Fiorucci, Pierre Poulain, Chantal Prévost, Martin Zacharias

**Affiliations:** 1 Computational Biology, School of Engineering and Science, Jacobs University Bremen, 28759 Bremen, Germany; 2 LBT, CNRS UPR 9080 and Université Paris Diderot – Paris 7, IBPC, 13 rue Pierre et Marie Curie, 75005 Paris, France; 3 DSIMB, Inserm UMR-S665, Université Paris Diderot – Paris 7, Institut National de la Transfusion Sanguine (INTS), 6 rue Alexandre Cabanel, 75015 Paris, France; 4 LCMBA, UMR-CNRS 6001, Faculté des Sciences, Université de Nice-Sophia Antipolis, 06108 Nice Cedex 2, France

## Abstract

**Background:**

Macromolecular docking is a challenging field of bioinformatics. Developing new algorithms is a slow process generally involving routine tasks that should be found in a robust library and not programmed from scratch for every new software application.

**Results:**

We present an object-oriented Python/C++ library to help the development of new docking methods. This library contains low-level routines like PDB-format manipulation functions as well as high-level tools for docking and analyzing results. We also illustrate the ease of use of this library with the detailed implementation of a 3-body docking procedure.

**Conclusion:**

The PTools library can handle molecules at coarse-grained or atomic resolution and allows users to rapidly develop new software. The library is already in use for protein-protein and protein-DNA docking with the ATTRACT program and for simulation analysis. This library is freely available under the GNU GPL license, together with detailed documentation.

## Background

Most biological processes in the cell involve macromolecules interacting with one or several partners [1]. Knowledge of the overall structures of these assemblies as well as the details of the interactions is essential for understanding the underlying biological mechanisms or for developing new therapeutic strategies. In spite of spectacular progress, the determination of the three-dimensional structure of large complexes at atomic resolution by means of X-ray crystallography or nuclear magnetic resonance spectroscopy remains a difficult task. Even in the case of binary complexes (two macromolecular partners), the number of available structures only represents a minor fraction of the complexes known to exist. Given the deficit of structural information on these assemblies and the increasing number of available structures for isolated proteins, computational modeling tools provide a promising approach to predicting structures of protein complexes. Docking methods are increasingly reliable and efficient for assembling macromolecular complexes when the partners do not present any large internal deformation. Numerous studies have been dedicated to protein-protein interactions [1] and the worldwide challenge "Critical Assessment of PRedicted Interactions" (CAPRI) [2-4] demonstrates the interest of the scientific community in this domain. The main challenges that need to be addressed for constructing macromolecular machineries concern the size and the number of the partners, and also their flexibility. A number of partners greater than two already leads to combinatorial problems [5] that are difficult to manage when searching the space in terms of relative rotations and translations. Very large partners make the search computationally costly.

Concerning flexibility, conformational adjustment induced upon association can lead to complete remodeling of the partner interfaces, thus making surface recognition inefficient when starting from the structure of the isolated partners. Several methodological approaches are being explored to overcome this particularly difficult problem [6-12], which must combine exploration of the macromolecule internal flexibility (thousands of degrees of freedom) and rapidity of the search. We have investigated two of these approaches, namely a normal mode approach that restricts the internal conformational search to privileged deformation directions [10] and a multi-copy approach that pre-generates ensembles of possible conformers to represent flexible protein parts [11,12]. The conformers are then attributed a weight that varies during the docking process. In addition to these methodological developments, we have developed coarse-grain models and associated force fields, directed to both proteins [13] and DNA [14], in order to allow the docking of large macromolecular systems. The level of graining is moderate, corresponding to four to five heavy atoms grouped together in each bead. This allows conservation of the main features of the surface geometry, which is essential for detection of surface complementarity. Our exploratory efforts also bear on the development of scoring functions that adequately account for the strength of protein-protein or protein-DNA association.

In order to develop methodological investigations as well as to optimize parameters, we needed a tool capable of performing and analyzing routine docking simulations, but that was also sufficiently flexible to allow easy testing and adding new functionalities in an efficient and rigorous fashion. For these reasons, we have developed the docking library PTools, which relies on a modular, object-oriented implementation based on Python/C++ coupling. Its multi-language object-oriented paradigm is shared with other libraries like MMTK [15] or the new EGAD library [16] indicating a convergence toward modular design.

PTools can handle coarse-grained as well as atomic macromolecular objects that can be compared or superposed for the purposes of analysis, or that can be docked using multiple energy minimizations in the coarse grained representation according to the ATTRACT protocol [13]. In this article, we present this library along with the principles that have guided its development. We expose the motivations for our choices in terms of programming and we provide several examples of its utilization for the docking problem. Finally, we illustrate the potentialities of our library for facilitating further developments, like testing new force fields or investigating docking algorithms. We detail how new methods can be implemented and tested in a case of a multi-protein docking strategy that avoids the problem of combinatorial explosion of possible start structures. The PTools library can be downloaded at .

## Implementation

### Design Goals

**Figure 1:**
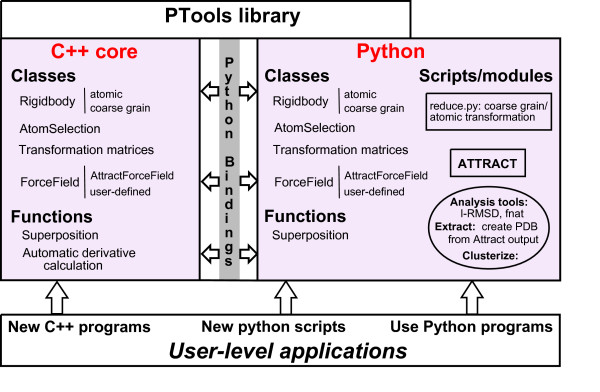
**Schematic representation of PTools architecture**. PTools C++ classes and functions (left) are in close correspondence with Python objects and functions (right) via Python bindings. The user can use PTools-based Python scripts delivered with the library and among them, ATTRACT or reduce.py, the translation script from atomic to coarse grain (right arrow). Analysis tools enclosed in a circle can be used as indepedent programs as well as Python modules. The user can also write his own scripts, or directly write C++ programs.

The PTools library has been designed in order to perform assembly tasks in an efficient way and to ease developments without sacrificing speed for correctness. We chose an object-oriented approach with a few free functions [17]. Figure 1 describes the library architecture. We detail below the reasons why we chose a Python/C++ solution.

#### Speed

Docking simulations require numerically intensive functions and speed was thus one of our concerns. We chose the C++ programming language to develop the core library since it allows the writing of both fast and flexible code. Indeed, C++ is fast because it is a statically typed, compiled language. C++ is also flexible because of many advanced features such as virtual functions, templates, the Standard Template Library, operator overloading, etc. Naturally some of these features (for instance virtual functions) come with some speed overhead, [18] but they were not used in the time-critical routines of the library.

#### Correctness

In the design of this library, large efforts were made to prevent errors or to catch them as soon as possible. With the use of standard C++ containers and smart pointers to hold dynamically allocated memory, we avoid most of the memory issues often encountered in software. Preprocessor macros are heavily used to make consistency checks in most of the functions (bond-checking for instance). For performance issues, these checks are disabled in release mode. In addition, core features are tested with unit tests. These tests guarantee that an expected behavior still works when adding new functionalities. Finally, runtime errors are propagated through C++ exceptions, preventing the programmer from simply ignoring an error return code. Furthermore, the library successfully passes valgrind's memory debugger [19] which detects various types of bugs like memory leaks or use of undefined values.

#### Ease of use

We also aimed for simple tasks to be easy to implement while complex ones should be as intuitive as possible. This principle has guided most of our design choices. For example, a single line of code is required to load a protein from a PDB file. Only one line is also needed to move a protein along a given translation vector, or to make a rotation. However, we also allow advanced users to access low-level methods. For instance, all properties of a protein single atom can be altered.

#### Ease of modification and extension

The object-oriented framework of the library simplifies modifications and extensions of the code. For example, all force fields are derived from a base abstract class requiring users to provide only two functions, one for energy calculation and the other one for its derivatives. To simplify the task of developing a new force field, derivative correctness can be automatically checked (see "Automatic derivative calculation"). A second-generation force field is under development and has already given promising results, especially for the difficult case of antigen-antibody complexes.

### Python Bindings

While this C++ library has been designed to be usable by pure C++ programs, the simulation software consists of Python scripts. This choice has been motivated by the fact that the Python programming language is increasingly used in the bioinformatics community [20]. Indeed, this language is reasonably easy to learn [21], easy to read with block indentations and also very comprehensive, with numerous additional modules such as command-line option parsing, matrix handling, multithreading, interactivity with others programs, etc. Finally, Python, as an interpreted language, does not require compilation after each modification thus allowing rapid and flexible development of new features.

#### Automatic generation of bindings

The Python C application programming interface (C-API) is the most natural and flexible way for interfacing C/C++ code with Python. However, this solution is time-consuming and error-prone since objects are manually interfaced from one language to the other. Furthermore, frequent developments and improvements in the code considerably complexify the Python bindings of the library. This justifies the requirement for both an efficient interface between C++ and Python and an automatic wrapping of the code.

The Boost Python library can be seen as a C++ wrapper for the Python C-API. By using sophisticated C++ mechanisms, it handles most of the housekeeping code like incrementing counters or translating C errors into Python exceptions. With the help of Boost.python, exposing a C++ class to Python is then as easy as providing the class name and all of its method names. However, the interface files still need one line for the class name, and one line for each member we want to expose. Keeping the interfacing code in sync with the library objects thus still requires significative investments.

To help in the creation of the interface file, we use a code generator called Py++ [22]. This Python module, with the help of few other programs, reads a C++ header file and automatically generates the correct code for exposing free functions and classes described within this header. A single line of Python code is required to wrap a C++ class with all its public methods.

### Toolbox

#### PDB I/O

Loading a PDB file into a Rigidbody object is extremely simple and requires a single line of code. In the following example in C++ the 1GC1.pdb file is loaded into the Rigidbody object prot. Then we select the chain A of the protein and write it into a new PDB file.

Rigidbody prot("1GC1.pdb");

AtomSelection selA = prot.SelectChainId("A");

Rigidbody chainA = selA.CreateRigid( );

WritePDB(chainA,"1GC1_A.pdb");

The equivalent Python code is:

prot = Rigidbody("1GC1.pdb")

selA = prot.SelectChainId("A")

chainA = selA.CreateRigid( )

WritePDB(chainA,"1GC1_A.pdb")

The similarities between both languages result in near-identical APIs for the library, the main difference in the example above being that the type of a new variable is not declared in Python. Further examples will be only given in C++.

The Rigidbody object contains a vector of atom objects and all atomic properties remain accessible and modifiable using low level methods. Here, as an example, we extract the third atom of the protein (indexed as 2 since the first atom is numbered 0) and modify its coordinates, its residue identifier and its name.

Atom atom = prot.CopyAtom(2);

Coord3D new_xyz = Coord3D(2.23,6.12,8.56);

atom.SetCoords(new_xyz);

atom.SetResidId(1);

atom.SetResidType("LEU");

#### Selection of atoms

The class *AtomSelection *implements a convenient method for selecting atoms from a protein or DNA molecule. User can filter atoms on properties like atom types, residue name, residue number, backbone or side chain. Selections can be combined using ensemble operators *AND*, *OR *and *NOT *which give full control over which atoms are included in an efficient and intuitive way.

As an example, the following code creates a selection containing non-C*α *atoms of residues 5–36 and 40–52 of the Rigidbody prot.

AtomSelection sel1 = prot.SelectResRange(5, 36);

AtomSelection sel2 = prot.SelectResRange(40, 52);

AtomSelection sel3 = prot.CA( )

AtomSelection result = (sel1 | sel2) & !sel3;

#### Rigid-body transformation tracking

Translations and rotations of molecules are internally stored into a 4 × 4 homogeneous coordinate matrix. Combined with a lazy evaluation of atom coordinates, this allows users to naturally express series of transformations without hurting performance. Indeed, when a user asks for a sequence of rotations and translations, only its associated matrix is computed. Cartesian coordinates are updated only on explicit request. An additional advantage of these matrices is the storage of docking results. Indeed, a typical docking simulation generates thousands of geometries (see ATTRACT protocol), and replacing final ligand coordinates by a matrix saves a lot of disk space. The following C++ code shows an example of a *π*/4 rotation of Rigidbody prot around axis (AB), followed by a translation:

#define PI 3.141592653

Coord3D ptA(3.0, 4.0, 5.0);

Coord3D ptB(12, -5, 2);

prot.ABRotate(ptA, ptB, PI/4.0);

Coord3D tr(6, 7, 8);

prot.Translate(tr);

The above code runs in constant time with respect to the number of atoms because, due to the lazy evaluation, only a 4 × 4 matrix has been modified.

#### Superposition

A Root Mean Square Deviation (RMSD) superposition algorithm [23] has been introduced into the library. With the help of selection methods, users can superpose two molecules in various ways provided that the two selections have the same size. The result of a superposition is an object which contains the RMSD after superposition and a 4 × 4 homogeneous matrix to be applied to the mobile element to obtain this calculated RMSD.

The following code shows a superposition of two Rigidbody objects prot1 and prot2 which have the same number of atoms.

Superpose_t sup = superpose(prot1, prot2);

double rmsd_best = sup.rmsd;

Matrix mat = sup.matrix;

Variable mat now contains the matrix that has to be applied to prot1 in order to minimize the RMSD between prot1 and prot2.

#### Helical parameters

The library also provides a function which translates a 4 × 4 matrix into a screw motion [24] (a combination of a rotation and a translation collinear to the rotation axis), which allows the reconstruction of a helical filament given two units. Helical parameters such as pitch and number of monomers per turn can also be extracted from a 4 × 4 matrix. The 4 × 4 matrix can be obtained from a superposition, or from the result of a docking process.

#### Minimizer

In the PTools library, we interfaced the L-BFGS minimizer written in FORTRAN by Jorge Nocedal [25,26]. L-BFGS is a limited-memory quasi-Newton minimizer used to solve nonlinear optimization problems. To compute the minimum of a multi-variable function it requires the gradient (but not the Hessian) of the objective function and accelerates the convergence by storing a low-rank approximation instead of the entire Hessian matrix.

#### Force fields

The BFGS minimizer requires the number of free variables describing the degrees of freedom of the system and access to both a function to minimize and its partial derivatives. The ForceField abstract base class is responsible for providing this information to the minimizer through virtual functions. Force fields can be implemented by deriving the ForceField class.

The PTools library contains by default the force field used by the docking program ATTRACT for protein-protein and protein-DNA docking. This force field applies to reduced protein and DNA representations. Reduced proteins are described by up to three pseudo-atoms per residue [13,27]. For DNA, each nucleotide is described by 5 to 6 beads, made of 3 to 5 heavy atoms [14]. This model assumes no internal energy evaluation since it has been developed for systematic rigid body docking. The effective interaction between two partners is the sum of a soft Lennard-Jones potential and an electrostatic potential. Both reduced model are compatible and show good performances in protein-DNA docking [14].

With the help of the PTools library, a new protein force field is currently under development with modifications in the protein backbone representation that allow a more realistic description of its polar character. Preliminary results look very promising especially for the ranking issue. We are also evaluating a modified Lennard-Jones scoring function with pairwise interaction terms (Fiorucci *et al*, in preparation).

#### Automatic derivative calculation

Minimizers, such as quasi-newton or conjugated gradient methods, require both a function to minimize and its derivatives. Usually energy functions used in molecular modeling are not trivial and errors may occur either during the determination of the derivative formula or during its implementation into the source code. In addition some minimizers may complain about inconsistent derivatives, while others may also return bad results. A possible approach to detect incorrect derivatives is to calculate numerical derivatives of a function *f *using the finite difference method. Here is the one dimensional formula:



However, the above formula is subject to roundoff errors which can affect even the first digits of the result. To prevent this well-known problem of roundoff errors [28,29], we integrated a C++ automatic differentiation tool provided by Pr. Martins and Peter Sturdza [29]. This tool uses the C++ ability to manipulate user-defined classes instead of built-in types with arithmetic operations. The new type, called *dbl*, has two double precision components: the first one is the value of the variable while the second is its derivative. Any arithmetic operation involving at least one *dbl *returns a *dbl *with both components accordingly set.

As an example, we may consider the following function, inspired by Sturdza et al. [29]:



The corresponding C++ function is:

dbl f (dbl x, dbl y){

return (exp(x)+y)/sqrt(pow(sin(x),3)

   +pow(cos(x),3));

}

Here is how to calculate both the function and its derivative at *x*_0 _= 1.5, *y*_0 _= 2.0:

dbl x0(1.5, 1);

dbl y0 = 2.0;

dbl result = f(x0, y0);

The first line sets the regular value of *x*_0 _to 1.5 and its *derivative *part to 1, meaning that *f *must be differenciated with respect to *x*_0_. Here *y*_0 _has a regular value of 2.0 and a null derivative part. The last line simply calls the function, and the result variable stores two values: the expected result and the derivative part of *f *with respect to *x*. A further call to *f *with dbl x0 = 1.5; dbl y0(2.0, 1); will return the derivative of *f *with respect to y.

Because this feature is only activated by a compilation flag in debug mode, it does not reduce speed or increase executable size in release mode.

#### Documentation

Extensive documentation is provided, with a tutorial describing every step from the compilation of the library source code to full protein-protein and protein-DNA docking simulations with ATTRACT. The C++ API is also automatically parsed by Doxygen [30] which generates a browsable documentation with an exhaustive description of every class and member function within the library. These reference pages may also be very useful for the users of the Python-side of the library since function names are conserved in the C++/Python binding.

## Methods and included docking tools

The Python tools presented in this section articulate the PTools C++ library functions for docking applications.

### ATTRACT

The ATTRACT docking program is implemented as a Python script using the PTools library. This script is also provided with the PTools package. The docking protocol of ATTRACT has already been described in previous publications [13,27]. Briefly, ATTRACT performs systematic docking without using any experimental data concerning the native complex. This algorithm relies on minimization of the interaction energy, the ligand (mobile partner) being placed at regular positions/orientations around the receptor surface (fixed partner) at a distance slightly larger than its biggest dimension. For each starting position, about 250 initial ligand orientations are generated. For each starting geometry, energy minimization (quasi-Newton minimizer) is performed using transitional and rotational degrees of freedom of the ligand. For instance, we carried out a rigid body docking simulation of bovine alpha-chymotrypsinogen A complexed with the pancreatic secretory trypsin inhibitor (PDB: 1CGI) using nearly 53000 different starting configurations of the bound form. The top-ranking solution is very close to the X-ray structure (Ligand-RMSD = 1.2 Å) and the simulation was achieved in 19 hours using a single core of an Intel Xeon Quad Core running at 1.86 GHz.

Different Python scripts are provided with the ATTRACT program to set up the input files needed by the ATTRACT docking script. First, the coarse-grained representation of the macromolecule is automatically generated by the reduce.py script. Second, translate.py defines ligand starting positions at a given distance from the receptor surface. It employs a slightly modified Shrake and Rupley method [31] and the density of the starting points around the receptor can be defined by the user. Finally, the ATTRACT input file contains all the specifications required to process the docking simulation (number of minimization steps, cutoff, etc.). Several minimizations (with decreasing cutoff) are necessary because the pairlist to calculate the interactions is only generated at the beginning of each minimization.

### Analysis scripts

To process and analyze the docking simulation outputs, a set of Python scripts based on PTools are also provided. These scripts are well adapted to the ATTRACT output format but may be used independently as well.

Our clustering algorithm, implemented in cluster.py, can rapidly group nearly identical structures without requiring a preselected number of desired clusters.

If the structure of the bound complex is known, some additional functions may be used to evaluate the quality of the predicted interfaces. In addition to the RMSD, interface RMSD (I-RMSD) and the fraction of contacts in native structure (*Fnat*) are of great help in assessing docking results [4].

### Parallel computing

Practically, a docking simulation consists of several independent energy minimizations. A single simulation can thus be split into smaller ones and run across a computer cluster. We used the Condor workload management system [32] to distribute our docking simulations on a 50 nodes cluster, with one job per starting point. The observed scaling is excellent. A docking simulation taking 2121 minutes on a single core can be achieved in 278 minutes when running on 8 identical nodes and 139 minutes for 16 identical cores. This corresponds to a speed-up factor of 7.6 for 8 cores and 15.3 for 16 cores. It is interesting to note that the 16 core simulation was exactly 2 times faster than the 8 core simulation.

## Results

As mentioned previously, one of the benefits of the library design is to allow rapid extensions of the docking tool. Here, we show how the library can be used to investigate methods for managing an arbitrary number of molecules, or in other words, to perform multiligand docking. Since the ATTRACT force field is able to deal with both protein-protein and protein-DNA complexes, this will open the way toward the assembly of various systems.

When the number of partners is greater than two, the systematic docking approach of ATTRACT cannot be used because of a combinatorial explosion [5] in the definition of starting points for minimization. Using PTools functionalities, we devised a strategy to overcome this limitation. The main idea is to limit the number of starting points for the 3-body docking simulations by combining high ranked solutions from the 2-body docking simulations.

More precisely, we investigate the 3-body problem with the following approach, described here for a test-case system formed by the globular head of the complement system protein C1q (PDB: 1PK6) [33]. We assume that one of the partners is known to interact with the two others, which is generally the case for 3-body systems that need to be assembled. In the following, the 3 partners are labeled units A, B and C, and unit A is taken as the reference for two systematic pairwise docking simulations with the other two units (B and C).

After a proper initialization step (including coarse grain reduction), one can perform the two 2-body docking simulations with ATTRACT.

$ Attract.py unitA.red unitB.red > AB.out

$ Attract.py unitA.red unitC.red > AC.out

In the above example, the .red filename suffix is used to easily distinguish reduced coordinates files from regular PDB files. More details about ATTRACT command line options are available in the tutorial provided with the PTools library.

From the clustered results of each 2-body docking simulation, we extract the 300 best candidates.

$ cluster.py AB.out unitB.red | head -300 > bestB.out

$ cluster.py AC.out unitC.red | head -300 > bestC.out

Each of the 300 × 300 possible combinations of dimer structures is used as docking start structure to generate complexes with the three partners. The command for extracting the AB docking prediction ranked 26th (translation number 128, rotation number 32, RMSD = 8.1 Å with respect to the structure of B in the crystal complex) and the AC docking prediction ranked 5th (translation number 73, rotation number 35, RMSD = 4.8 Å with respect to the native structure of C) are as follows:

$ Extract.py attractAB.out unitB.red 128 32 > B_128_32.red

$ Extract.py attractAC.out unitC.red 73 35 > C_73_35.red

At this point, structures with too many clashes are discarded and we optimize the remaining structures by a series of energy minimization steps where the reference unit (A) is kept fixed but the other two partners (units B and C) are allowed to move freely in translation and rotation.

#### Python script: minimize3body.py #####

from ptools import *

A = AttractRigidbody("A.red")

B = AttractRigidbody("B_128_32.red")

C = AttractRigidbody("C_73_35.red")

A.setRotation(False) # don't allow rotations and

A.setTranslation(False) # translations for unit A

#loads a forcefield with a cutoff of 12A

forcefield = AttractForceField1("aminon.par", 12.0)

#populates the simulation box

forcefield.AddLigand(A)

forcefield.AddLigand(B)

forcefield.AddLigand(C)

#creates a minimizer instance:

lbfgs = Lbfgs(forcefield)

lbfgs.minimize(50) # minimizes for at most 50 steps

An arbitrary number of molecules can be added to the simulation with the AddLigand method.

**Figure 2:**
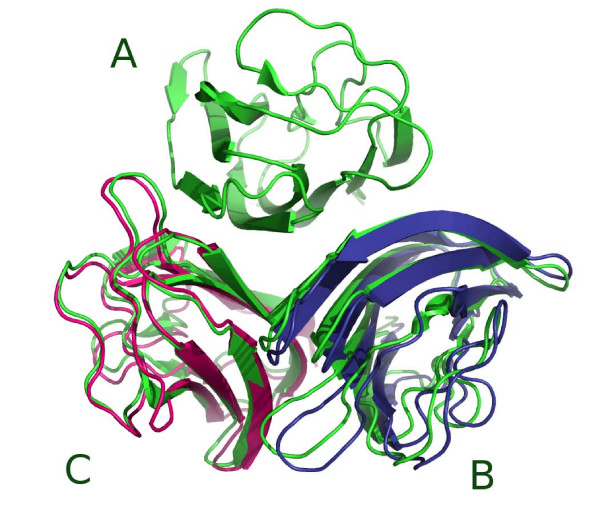
**C1q system**. (PDB: 1PK6) The crystallographic system is represented in green. Units B (in blue) and C (in magenta) were obtained after the 3-body docking strategy (see text).

We tried to keep the use of the different classes as natural as possible from the programmer's perspective. After minimization, the lbfgs object contains the energy of the minimized system as well as the effective values of the free variable set. The minimizer also stores the different states of the system for each minimization step. This permits the generation of movies with visualization software like PyMol or VMD to inspect a simulation (as described in the tutorial). In spirit this strategy for multiligand docking is similar to the approach introduced by Inbar et al. [5]. However, in their approach high ranking dimers are combined rigidly into trimeric complexes without the possibility to readjust the already formed pairwise (dimeric) complexes. In our test, the 3-body readjustment docking step with two mobile partners (units B and C) resulted in a very significant improvement of the deviation of the structure with respect to the native complex. For example, the RMSD of B compared to its reference position decreases from 8.1 Å to 2.8 Å in the best ranking case (see Fig. 2).

Once the strategy has been sufficiently tested and optimized, the instructions for the multiligand docking can be easily gathered into a new script. This time, the script files cluster.py and Extract.py will be used as regular python modules rather than standalone programs.

## Discussion

### Other Tools

PTools is not the only available tool for molecular modeling, not even for molecular docking. Before discussing the multiple advantages of PTools, we will describe other available tools.

MMTK is an open source molecular modeling library written by Konrad Hinsen [15]. This library is written in Python, with some computationally intensive routines written in C. MMTK comes with functions and classes for molecular modeling and molecular dynamics, using the Amber force field. A protein-protein docking program, pyDock [34] has been built on top of this library.

The EGAD library [16] is a C++ object-oriented library dedicated to protein modeling. It provides many tools from basic molecular manipulations to side chain refinements with the OPLS-AA forcefield. By design and philosophy, PTools is comparable to EGAD. Yet they still differ on certain points. EGAD is focused on protein design rather than molecular docking and it uses all-atom representations. We found out that each project may benefit from the other, which could be the subject of future work.

HADDOCK [35] is a suite of programs dedicated to docking. It is built on top of CNS, a molecular modeling library designed for crystallography and NMR applications. This library defines its own scripting language.

Biskit [36] is a molecular modeling library written in Python for various purposes. High-level applications like docking, homology modeling and molecular dynamics are performed using external programs and other software can be easily added.

Some molecular modeling programs which are not designed for docking purposes can also be cited. MODELLER [37] is a closed-source suite of tools for homology modeling with a Python interface. While this program is not intended for protein-protein docking it can be useful to generate starting structures for docking.

MGLTools is a Python library for visualization preparation and analysis of molecular structures. It is coupled to Autodock for preparing inputs and analyzing outputs.

### PTools Advantages

The goal of PTools is to provide a scaffold for development and implementation of new molecular modeling methods. In this purpose, the most interesting points of this library are:

• *Generality*: The PTools library can handle both all-atom and coarse grained representations. Tools are provided for translating from all-atom to reduced models and for retrieving all-atom coordinates from docking simulations.

• *Flexibility*: PTools has been designed as a library and therefore eases the development of new applications, in contrast to monolithic programs.

• *Language choice*: The programmer can either use the PTools library as a pure C++ library or as a Python module. We also provide a very efficient way to generate new Python bindings. Most C++ classes can be interfaced using a single, simple line of code, without any knowledge of the Python C-API.

• *Easy to extend*: Our automatic check of first derivatives is much more precise than the finite difference method and can therefore help for the design of new forcefields.

• *Freely available*: This library is open-source and can be freely studied, modified and distributed provided that modifications remain open-source. We also do not rely on any proprietary external dependency.

• *Well documented*: A tutorial describing installation and use of docking tools is distributed along with the library. Automatic parsing of the source code provides a valuable and always up-to-date documentation for developers.

• *Cross-platform*: This library has been successfully compiled and tested on various linux distributions and Mac OSX platform. It should compile on Windows as well with very few modifications, probably limited to the compilation environment. Feedback from Windows users is welcome and we will update our documentation and compilations tools in order to take their experience into account.

## Conclusion

We present in this article a new library for molecular modeling and docking that provides the modeling community a user-friendly way to manipulate both coarse-grained and atomic representations of macromolecules. Its object-oriented design allows rapid development of new features. The library can be used as a pure C++ library or as a Python module. Binding PTools with Python brings to the developer a higher level set of functions and modules that help improving overall code quality, as well as favoring easy testing and implementation of new docking strategies. We have illustrated this potentiality with the investigation of a new methodological strategy aiming at docking an arbitrary number of proteins. Tests and applications of the multi-protein docking approach to bound and unbound systems will be subject of future work. Future improvements of the PTools library will include the possibility to account for conformational flexibility of the association partners during docking, using methods that are presently being explored using the ATTRACT program [10,12].

## Availability and requirements

• Project Name: PTools (library) and ATTRACT (docking program)

• Project Home Page: 

• Operating system(s): Linux, MacOSX

• Programming Language: C++/Python

• Other requirements: standard programs and libraries such as scons, gccxml, pygccxml, py++, the Boost libraries (a step-by-step installation guide is provided within the tutorial)

• License: GNU GPL version 3

• Restrictions on use: only those of the GPL v3 (see the COPYING file in the source directory)

## Authors' contributions

AS designed, wrote and tested the C++ library, made the Python bindings, ported the original ATTRACT code to C++ and Python, and performed the 3-body docking experiments. PP and SF wrote code for the C++ library and Python scripts and tested the library. PP wrote the library tutorial. MZ wrote the original ATTRACT code. CP and MZ supervised the multiligand experiments. All authors participated in the writing of the manuscript.
